# Functional genomics of human brain development and implications for autism spectrum disorders

**DOI:** 10.1038/tp.2015.153

**Published:** 2015-10-27

**Authors:** M N Ziats, L P Grosvenor, O M Rennert

**Affiliations:** 1 Laboratory of Clinical and Developmental Genomics, National Institute of Child Health and Human Development, National Institutes of Health, Bethesda, MD, USA; 2 University of Cambridge, Robinson College, Cambridgeshire, UK; 3 Baylor College of Medicine MSTP, One Baylor Plaza, Houston, TX, USA; 4 Pediatrics and Developmental Neuroscience Branch, National Institute of Mental Health, National Institutes of Health, Bethesda, MD, USA

## Abstract

Transcription of the inherited DNA sequence into copies of messenger RNA is the most fundamental process by which the genome functions to guide development. Encoded sequence information, inherited epigenetic marks and environmental influences all converge at the level of mRNA gene expression to allow for cell-type-specific, tissue-specific, spatial and temporal patterns of expression. Thus, the transcriptome represents a complex interplay between inherited genomic structure, dynamic experiential demands and external signals. This property makes transcriptome studies uniquely positioned to provide insight into complex genetic–epigenetic–environmental processes such as human brain development, and disorders with non-Mendelian genetic etiologies such as autism spectrum disorders. In this review, we describe recent studies exploring the unique functional genomics profile of the human brain during neurodevelopment. We then highlight two emerging areas of research with great potential to increase our understanding of functional neurogenomics—non-coding RNA expression and gene interaction networks. Finally, we review previous functional genomics studies of autism spectrum disorder in this context, and discuss how investigations at the level of functional genomics are beginning to identify convergent molecular mechanisms underlying this genetically heterogeneous disorder.

## Human brain gene expression

**Figure 1:**
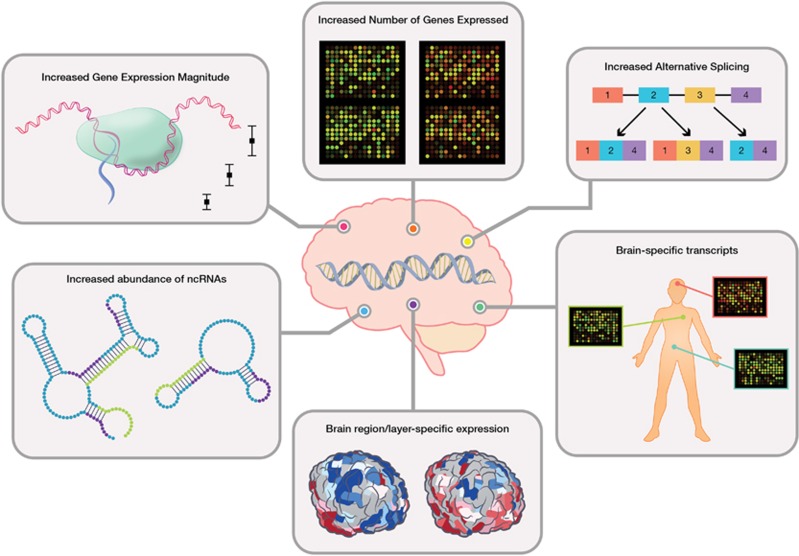
Summary of unique functional genomic properties of the human brain. Unique human-specific RNA expression properties discussed in the text are diagrammed. ncRNA, non-coding RNA.

Across human development, a single gene can be expressed in multiple isoforms and varying orders of magnitude depending on the particular developmental context; that is, the tissue, stage of development and local or long-distance signaling mechanisms being received by the cell or tissue. Therefore, in order to understand how a gene may contribute to a developmental disorder, it is critical to first understand its normal expression pattern and function in the appropriate tissue during the developmental time window of interest. This is especially important for human neurodevelopment, as human brain gene expression in particular has been demonstrated to be unique in a number of ways (Figure 1). The consistent reports describing the uniqueness of the human neurodevelopmental transcriptome underscore the need to study genes implicated in neurodevelopmental disorders in their appropriate functional genomic context, in order to understand their role in disease more accurately.

Compared with other species, human brains express messenger RNA (mRNA) transcripts at much higher levels and with much greater complexity. For instance, comparisons of human brain gene expression with both mice^1, 2^ and primates^3, 4^ has demonstrated that most of the differentially expressed genes between the species are upregulated in humans, but this phenomena is not apparent in other tissues. In addition, the human brain expresses ~85% of all genes encoded in the human genome at some point during development,^5^ which is greater than any other human tissue type. It is hypothesized that this increased level of gene expression is at least partially responsible for the higher level of neuronal activity and overall cognitive function of humans.

Within humans specifically, the brain also displays a distinct gene expression profile compared with other tissues. Using both array^6^ and sequencing-based techniques,^7^ the brain has been shown to have higher expression levels and greater transcriptome complexity than most other human tissue and cell and tissue types, with perhaps the notable exception of sperm.^8, 9^ In particular, the human brain transcriptome displays a high level of alternatively spliced transcripts,^10, 11, 12^ and the set of isoforms produced in brain differs considerably from other tissue types.^6, 10^ In addition, the human brain transcriptome also contains a distinctively high number of non-coding RNAs (ncRNAs). In fact, the brain displays the greatest abundance of transcribed ncRNAs among all tissues.^13^ Both short ncRNAs, such as microRNAs (miRNAs) and PIWI-interacting RNAs, and long ncRNAs (lncRNAs) are highly enriched in human brain tissue.^14, 15, 16, 17, 18^ As ncRNAs are becoming increasingly recognized as important regulatory elements in genome processing during neurodevelopment, and in the pathogenesis of neurodevelopmental disorders,^19^ their abundance in the brain further highlights the uniqueness of neurodevelopmental functional genomics.

Although within a given brain region the human transcriptome has been shown to be incredibly complex, it is also of importance to consider the relationship among different anatomical regions of the brain, as ‘disconnectivity' between disparate brain regions is thought to underlie a number of neurodevelopmental syndromes such as autism spectrum disorders (ASD).^20, 21^ Perhaps unsurprisingly, there is strong evidence that distinct regions of the human brain have distinct gene expression profiles, and animal studies have suggested that this variation is related to both structural and functional differences.^19^ For instance, a microarray study of 20 distinct brain and spinal cord sites showed that expression profiles can cluster samples from different donors by anatomical origin, and that some anatomical regions have up to 2000 region-specific genes.^22^ Multiple studies have shown that the cerebellum contains the most unique gene expression pattern compared with other brain structures,^2, 22, 23^ which is of consequence to the neurodevelopmental disorder autism in particular, as this region has been consistently implicated in its pathogenesis.^24^ Even within the neocortex alone, different cortical layers each express a distinct profile of mRNA transcripts.^25^ Further highlighting the importance of these region- and layer-specific expression properties are a number of reports that have shown that gene expression differences between any two brain regions within one individual are more pronounced than are gene expression differences between the same brain region of two different individuals.^4, 23, 26^

Taken together, these findings highlight the importance of understanding human psychiatric and neurodevelopmental disorders in the context of human brain gene expression specifically, as it is possible that animal, cellular and other models do not recapitulate human brain mRNA and ncRNA expression patterns with the appropriate level of fidelity. Although animal and cellular studies have and will continue to play an invaluable role in understanding how ncRNAs mechanistically influence gene expression and other processes at the molecular level, the identification of ncRNAs of relevance to neurodevelopmental disorders is most likely to come from post-mortem brain tissue studies of these diseases. Moreover, evidence is accumulating that suggests gene expression patterns within the human brain vary considerably across developmental time, and therefore temporal patterns of gene expression are an important consideration for neurodevelopmental disorders with childhood onset.

## Changes in gene expression during human neurodevelopment

The developing human brain grows remarkably fast—the weight of a newborn's brain is ~25% of its adult weight, but within 2 years, it nearly reaches adult size.^27^ During this time, the brain grows mainly through glial multiplication, myelination, formation of new synaptic connections and pruning of unused synaptic connections. Although the human brain continues to mature up to the age of 25,^28^ the greatest changes occur during infancy and early childhood. Coincidentally, most neurodevelopmental disorders become clinically recognizable around these ages.

Underlying these marked early changes in gross brain development are complex and dynamic broad patterns of gene expression change, which have only recently begun to be understood. The most comprehensive study to date of the developing human brain transcriptome documented that genome-wide patterns of expression correspond closely to the major stages of clinical development (namely prenatal, early infancy, childhood, adolescence and adulthood), and that the molecular profile of these stages are distinct from each other.^5^ The most striking observation was that the greatest shift in gene expression occurs around the period of birth, when almost 60% of genes change their expression patterns in the neocortex. Other studies have demonstrated similar changes, and have shown that many of the genes identified during this shift are involved in cortical development and higher-order cognitive functioning.^29, 30^

The microarray study by Kang *et al.*,^5^ which assessed neurologically normal donor brains spanning the 2nd trimester through adulthood, also demonstrated that after infancy the number of genes whose expression profile changes in the neocortex decreases markedly to ~9% of expressed genes between infancy and adolescence, and less than 1% of genes between adolescence and adulthood. Functional annotation of these gene sets further revealed that genes expressed very early in prenatal development are highly related to the processes of cell differentiation, proliferation and migration, whereas genes expressed later in gestation are more related to synaptogenesis—suggesting that time-period-specific gene expression patterns drive cell-level developmental programs. Again, these findings highlight the importance of assessing candidate disease genes during the appropriate developmental time window, in order to gain the most relevant insight into their neurodevelopmental functions.

In addition to the greatest number of genes shifting their expression trajectory shortly after birth, the changes in gene expression in early postnatal life also have greater amplitudes of change than during other times.^31, 32, 33^ In fact, it was shown that many genes actually completely reverse their expression trajectory in early life, mostly shifting from a trend of increasing expression in fetal life and infancy to decreasing expression beginning in childhood.^31^ Moreover, as the brain begins to mature, the gene expression profile within each anatomical region becomes more similar to other brain regions (with the notable exception of the cerebellum), suggesting that most of the genetically encoded region-specific development is completed early in life. Interestingly, these broad gene expression patterns appear to reverse themselves in older age, at least in the prefrontal cortex.^33^

## Non-coding RNAs in human brain development

Since the advent of high-throughput, unbiased, genome-wide expression arrays and sequencing platforms, the recognition that the genome is pervasively transcribed at loci that do not encode for protein has become well recognized. Although originally considered to represent evolutionary ‘noise,' the non-coding component of the transcriptome has been increasingly shown to regulate the genomic landscape though a myriad of mechanisms, and as such are increasingly being recognized as important modulators of gene expression.^34^ Likewise, they are also beginning to be implicated in neurological disease.

NcRNAs can modulate transcription in a number of ways, such as interacting with DNA to induce methylation or histone modifications, recruiting transcription factors to promoters and modulating the three-dimensional architecture of chromosomes in the nucleus.^35, 36^ They can also bind to other RNA molecules, especially mRNAs with complementary sequences, to inhibit translation through RNA degradation, or they can act as ‘sponges' and thereby dilute the effect of mRNAs or other ncRNAs.^37^ Conversely, they can increase the rate of translation by acting as molecular stabilizing scaffolds, catalyze protein–protein interactions by linking otherwise scarce proteins together and participate in cellular trafficking of RNA-binding proteins.^38^ In addition, ncRNAs have even been shown to participate in intercellular communication by helping transport cargo between adjacent cells.^39, 40^

The increasing recognition of this important layer of transcriptome information is perhaps most important in the brain, where it has been shown that lncRNAs in particular are abundantly expressed in brain tissue,^41^ and that greatest abundance of ncRNAs appears to exist in brain (and perhaps testis) as compared with other tissue types.^42, 43^ Furthermore, there is strong evidence that ncRNAs had a critical role in the evolution of human brain structure and function. For instance, the fastest evolving regions of the primate genome are sequences that are transcribed into ncRNAs, and it has been shown that these particular ncRNAs are primarily involved in regulating neurodevelopmental genes.^44^ Similarly, regulation of transcription through RNA editing (which is particularly frequent among ncRNAs) has undergone a significant evolutionary expansion in higher primates and humans.^45^

This expanding inventory of ncRNAs, and their increasing functional and regulatory activities in humans, appear to have an important role in neurodevelopment and neuropsychiatric diseases. In particular, miRNAs and lncRNAs are the two most understood types of regulatory RNAs, and have recently been implicated in a number of neurodevelopmental disorders including ASD.

## MicroRNAs

The best studied of the ncRNAs are miRNAs, which mainly function to repress translation post-transcriptionally through the RNA interference mechanism. The miRNA family includes a variety of precursor RNA molecules that are classified mainly on their genomic origin, such as endogenous small interfering RNAs and PIWI-interacting RNAs. Despite their origin, all classes of small RNAs are quickly processed after transcription into their mature form, which for miRNAs is typically a 20–23-nucleotide, single-stranded molecule.^46^

In brief, the RNA interference mechanism begins when precursor miRNAs are transcribed in their entirety from the genomic DNA, processed by an enzyme complex known as DROSHA, exported to the cytoplasm and cleaved by the ribonuclease DICER into their mature form. At this point, they bind to a class of proteins termed Argonautes, and are then incorporated into a larger multi-protein complex termed the RNA-induced silence complex. The RNA-induced silence complex is guided to mRNAs that are complementary to the associated miRNA, leading to a repression of translation or overt degradation of the mRNA transcript.^47^

Importantly, a single miRNA can target—and therefore regulate—many mRNAs because of their short sequence, their preferential binding to 3′-untranslated regions and their imperfect complementary binding to cognate sequences.^48^ Conversely, it has been shown that individual mRNAs are often targeted by multiple miRNAs. Therefore, miRNA–mRNA interactions alone can increase the complexity of gene expression regulation by orders of magnitude. Furthermore, miRNAs are known to target other ncRNAs in addition to protein-coding mRNAs.^49^ The critical implication of these insights is that a single miRNA has the ability to modulate entire transcriptional networks, and therefore the mis-expression of a single miRNA has the potential to disrupt the proper expression of entire suites of genes.

Recent studies have demonstrated the importance of miRNAs in human brain evolution, cellular development, experience-dependent plasticity and in neuropsychiatric disorders. A large number of miRNAs exhibit species-specific expression patterns, are conserved only in primates and/or humans or are exclusively expressed in brain—providing strong evidence for their role in human-specific brain functions and disorders.^33, 50^ For example, an analysis of human, chimpanzee and macaque prefrontal cortex and cerebellum showed a substantial degree of divergence in their miRNA expression patterns.^51^

In addition, important roles for miRNAs in neural stem cell maintenance and differentiation have been established through a number of studies that have identified and characterized individual miRNAs of interest. For instance, multiple studies have shown that DICER knockout animals display a host of neurodevelopmental defects, including abnormal brain size, structural defects and improper formation of synapses.^52, 53, 54^ One particular miRNA, miR-9, has been studied extensively for its role in developmental patterning and cell migration, where it has been shown to be critical for neural stem cell self-renewal,^55^ production of some of the earliest neurons in the developing telencephalon and cortical laminization.^56^ Cellular studies of pluripotency have demonstrated that introduction of particular miRNAs can reprogram human skin fibroblasts into neuronal-like cells,^57^ and that this mechanism likely involves the central nature of these miRNAs in canonical transcriptional networks, which are known to guide neural cell fate decisions.^58^ In addition to neurogenesis, miRNAs have also been shown to regulate gliogenesis, in particular the formation of oligodendrocytes and astrocytes.^59, 60^

In addition to their role in individual cellular-level functions, miRNAs have been shown to contribute to synaptogenesis and experience-dependent plasticity—both functions thought to underlie complex human behavior, and hypothesized to be disrupted in ASD. For example, specific miRNAs are enriched in the synaptic nerve terminals of axons,^61^ and are upregulated in expression in the hippocampus following memory tasks in mice.^62^ In *Drosophila melanogaster*, knockout of subcomponents of the DICER complex results in synaptic transmission defects, but no overt brain structural abnormalities,^63^ and in mice result in dendritic spine malformations^53^ and impaired synaptic transmission.^64^ Remarkably, a complete absence of mature miRNAs due to DICER knockout affects memory and learning in mice.^65^

Finally, miRNAs have recently been recognized as contributing to human neurologic disease. Inherited variation in DNA encoding for miRNAs or their recognition sites has been linked to a number of disorders including schizophrenia.^66^ Dysregulated expression of miRNAs has been demonstrated in brain tumors,^67^ Parkinson's disease^68^ and Tourette's syndrome.^69^ Expression of miRNAs in normal human developing brain appears to be highly brain-region-, developmental-stage-, and even sex specific, and the putative target genes of the differentially expressed miRNAs were found to be highly related to known neurodevelopmental disorder risk genes.^70^

However, despite these substantial observations suggesting that miRNAs are critical regulators of neurodevelopmental transcriptional networks and are often disrupted in neurologic diseases, only a few small studies have attempted to profile miRNA expression levels in neurodevelopmental disorders such as ASD (reviewed below). Thus, there is a need for a comprehensive assessment of miRNAs in post-mortem brain tissue from individuals affected by neurodevelopmental disorders.

## Long non-coding RNAs

In contrast to miRNAs, which are short sequences with well-defined functions in post-transcriptional regulation, lncRNAs represent a novel class of transcripts whose function in brain development remains poorly understood. LncRNAs are defined as RNAs greater than 200 nucleotides in length (as compared with ~21–23 nucleotide length of miRNAs), which do not encode for protein, or lack an appreciable reading frame. LncRNAs undergo post-transcriptional processing similar to other RNAs, providing the first hint at their functional importance. For instance, some lncRNAs are modified to include a 5′-methyl cap and undergo 3′-polyadenlyation.^71, 72^ However, unlike miRNAs, lncRNAs are generally poorly conserved evolutionarily and as such, elucidation of their functional roles has relied more upon expression analysis and individual functional studies than on comparative genomic interpretations.

Although originally thought to be evolutionary byproducts of ‘junk DNA,' lncRNAs have been shown to be involved in major mechanisms of gene expression regulation, such as targeting transcription factors, initiating chromatin remodeling, directing methylation complexes and blocking nearby transcription.^35^ Moreover, pervasive transcription of lncRNAs has been demonstrated to occur in both a temporally and spatially regulated manner during development,^73^ with the central nervous system displaying the greatest abundance of transcribed lncRNAs.^41, 42, 43^

Recently, it has been demonstrated that individual lncRNAs have important regulatory roles in the spatial–temporal control of gene expression in the brain. One of the first studies to demonstrate this explored RNA expression from mouse *in situ* hybridization data, and the authors demonstrated that most lncRNAs examined were localized to specific cell types, subcellular compartments or neuroanatomical regions.^41^ This work provided some of the first large-scale evidence that lncRNAs may have specific functions in their capacity as RNAs alone in the mammalian brain. Subsequently, a number of studies employing both whole-genome and individual candidate lncRNA assessment have begun to expose the importance of lncRNAs to the regulation of the developing brain transcriptome. For example, individual lncRNAs are upregulated in response to neural activity and synaptogenesis.^74, 75^ They have also been implicated in neuronal differentiation; for instance, the lncRNA *Evf2* recruits a number of important neurodevelopmental transcription factors (such as *Mecp2*, the *DLX* family and *GAD1*) to their target genes in GABA-ergic interneurons, and *Evf2* knockout mice have reduced interneuron cell numbers.^76^ Transcriptome studies of brain tissue have also begun to characterize the entire landscape of lncRNAs during neurodevelopment. It was shown that lncRNAs are differentially expressed across layers of the mouse neocortex,^77^ and that those expressed in brain are preferentially located in genomic regions containing critical neurodevelopmental genes.^16^

LncRNAs have also been found to be abnormally expressed in a number of neurologic disorders. The first rigorous demonstration of the mechanistic function of a lncRNA in human neuronal activity and subsequent dysfunctional mechanism in disease was recently described for the lncRNA *Gomafu*, where the authors showed that *Gomafu* binds directly to the splicing factors QKI and SRSF1, and dysregulation of *Gomafu* expression leads to alternative splicing patterns that resemble those observed in SZ post-mortem brain tissue.^78^ Another example is the lncRNA *BACE1-AS.* This lncRNA is an antisense transcript of the beta-secretase-1 gene locus, which is implicated in the generation of beta-amyloid plaques in Alzheimer's disease.^79^ The *BACE1-AS* lncRNA extensively regulates the level of *BACE1*, and therefore can directly affect the level of beta-amyloid plaque accumulation.^80^ Other lncRNAs have been implicated in the neurodevelopmental disorder Angelman's syndrome, which shares many features with ASD.^81, 82^

Therefore, lncRNAs appear to have a critical role in modulating gene expression in the developing brain, and are increasingly implicated in neurological disorders. Their potentially numerous regulatory mechanisms, and largely overlooked sequence variation in disease, makes them an important class of candidate molecules to consider in neurodevelopmental disorders with complex genetics, such as ASD.

## Gene networks in the developing human brain

Although assessing non-coding regions of the genome is an important approach to comprehensively understanding the complex functional genomics of human brain development and neurodevelopmental disorders, it is equally important to consider how disparate genomic elements may work in concert with each other to produce biological effects that are emergent only after their interaction. The study of genetic interactions can be done by modeling large gene sets as networks of interacting nodes and edges, allowing for a statistical assessment of relationships among and between genes, as opposed to the study of individual genes themselves. Such approaches are particularly important in complex genetic syndromes such as ASD, as genome-wide association studies have consistently demonstrated that most individual variants in ASD have only very small effects by themselves.

The study of gene networks is a subset of the field of network science that applies mathematical and statistical principles to biological systems. A biological network is a system of individual biologic components that interact with each other in a structured, non-random manner, such that properties of the network as a whole emerge that are not apparent by studying the individual components in isolation. Biological networks have been identified at levels spanning molecular,^83^ cellular,^84^ organ system^85^ and even inter-individual relationships.^86^

**Figure 2:**
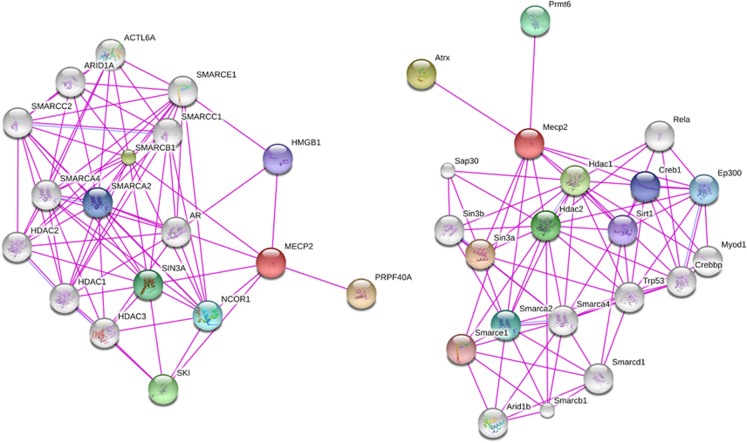
Examples of a protein interaction networks. The properties of networks as a whole can become apparent, which would not be appreciated by studying individual genes. For example, the network on the left represents known 2nd-degree protein–protein interactions with the gene *Mecp2* in humans, whereas the network on the right represents the known *Mecp2* interactions in mice. As can be seen, the human network (left) is much more densely connected, suggesting *Mecp2* has more known interactions in humans. More rigorous methods are available to quantify the degree of connectedness of such networks. Networks in this figure were created with String v9.05 (http://string-db.org/).

An interaction network consists of nodes and edges. In gene networks, nodes represent discrete genes and edges represent a biological relationship or connection between nodes (Figure 2). Depending on the interaction being studied, edges can represent many relationships such as known protein–protein binding, correlations of expression levels between genes, or any other metric that can be measured in all nodes and have putative biological relevance to how the system works. As most gene networks are incredibly large (thousands of nodes and millions of possible edges), the study of biological networks relies on a number of mathematical principles adopted from network theory and statistics that allows for the incredible complexity of large networks to be summarized, quantified and visualized in order to more easily infer biological meaning.

A genetic interaction occurs when an unexpected phenotype emerges from the combination of two or more interacting genes. This phenomenon is widely pervasive in genetics and has been recognized for decades. For instance, the phenomenon of synthetic lethality occurs when two genetic mutations, which by themselves cause no effect, are both present in the same gene and their presence together results in a defective protein product.^87^ Furthermore, genetic interactions appear to be ubiquitous throughout the genome.^88^ However, predicting how independent genes combine with each other to create emergent properties is not straightforward. This is especially true in non-model organism systems, such as the human brain, where it is impossible to manipulate individual genes, and therefore researchers must rely only on observational measures such as gene and protein expression levels.

One validated approach to integrate heterogeneous gene sets, in order to uncover shared molecular mechanisms, is through the analysis of gene co-expression patterns, which invoke the guilt-by-association heuristic that is pervasive in genomics research.^89, 90^ Several studies have demonstrated that genes with similar brain co-expression patterns are likely to function together in common cellular pathways.^91, 92^ These transcriptional co-expression relationships are particularly relevant to neurodevelopment, as the precise regulation of gene expression across brain regions at different ages instructs the exquisite specialization and connectivity within the brain. For instance, if two genes are expressed with similar patterns (that is, they have a similar magnitude and direction of expression change across developmental time), they would have a higher correlation than two genes whose expression appears to be randomly related to one another. In this network, edges would link genes with similar expression profiles, whereas unrelated genes would not share an edge. Defining edges between genes in this way allows the conclusion that the two nodes share related biological function, and can be used to derive and study large-scale genetic interaction networks.

Another widely used approach to infer interaction networks is to draw upon experimentally determined protein–protein interactions. Protein–protein interaction networks are perhaps the best-characterized networks in all of biology, and many well-curated experimental data sets containing detailed interaction information exist. Studies have demonstrated that protein interaction networks are conserved evolutionarily,^93^ and that proteins in the network with high degrees of connectedness are more important for organismal survival and fitness than are networks with lesser connectivity.^94^ This suggests that information on the importance of individual genes/proteins in a network can be inferred by studying the overall structure of the network as a whole. Such an approach could be particularly valuable in disorders such as ASD, where there are many implicated genes, but the relative importance of each to the pathophysiology of the disorder is unclear.

As neurodevelopmental disorders such as autism are believed to result from functional aberrations within brain regions and/or disruption of inter-regional connectivity between regions,^20^ investigating the gene expression profiles of autism candidate genes across brain regions and throughout normal human neurodevelopment may provide insight into the complex functional genomics of this neurodevelopmental disorder. Furthermore, as the genetic heterogeneity of ASD continues to increase as more sequencing and association studies are performed, prioritizing candidate genes through their location in interaction networks is an important undertaking.

## Functional genomics studies of ASD

Gene expression dynamics in early human brain development are clearly both spatially and temporally specific. This suggests not only that they are highly regulated, but that different genes and gene networks will have dynamic expression throughout space and time. Despite this increasingly recognized property of human neurodevelopmental genomics, until recently few studies of autism candidate genes have considered their expression and function in early human brain development. Furthermore, the molecular regulators of brain mRNA expression, such as ncRNAs, have not been extensively characterized in the developing human brain, and their potential involvement in ASD is only beginning to be explored. Accordingly, an understanding of the functional genomics of autism, the genetic interaction networks that ASD candidate genes participate in and the potential ncRNA regulators of these genes, represent important unresolved avenues of research.

## Gene expression studies in ASD

The majority of gene expression studies in autism have been performed in peripheral blood lymphocytes;^95^ however, as only approximately half of the genes expressed in brain are also expressed in lymphocytes,^96^ it is critically important that autistic brain tissue be assessed directly. The few genome-wide expression profiling studies in autistic brain tissue have repeatedly identified a number of functions that appear to be disrupted in autistic brain. The first microarray study of autistic brain tissue assessed post-mortem cerebellum and demonstrated dysregulation of AMPA receptor subunits in ASD.^97^ Subsequently, Garbett *et al.*^98^ analyzed genome-wide microarray expression profiles from six autistic temporal cortices and six controls, and their results suggested an upregulation of genes involved in immune and inflammatory processes with a concurrent downregulation of genes involved in neurodevelopment. The largest sample size assessed by microarray analysis to date studied three separate brain regions (frontal cortex, temporal cortex and cerebellum) from 19 autistic cases and 17 controls.^99^ This work also demonstrated an upregulation of genes with known immune function and a concurrent downregulation of genes involved with the synapse. Importantly, there was a large degree of overlap in the genes identified between the studies by Garbett *et al.*^98^ and Voineagu *et al.*,^99^ even though they assessed different cohorts of donor brains. More recently, a study of the autistic prefrontal cortex across a large age span demonstrated dysregulation in pathways governing cell number, cortical patterning and differentiation in the young autistic prefrontal cortex, but in contrast found dysregulation of signaling and repair pathways in adult autistic brain tissue.^100^ Another recent study of autistic cerebellar and occipital brain regions demonstrated significant gene expression abnormalities in mitochondrial oxidative phosphorylation and protein translation pathways.^101^

Although the number of studies assessing gene expression in autistic brain are still small, already there appears to be a growing body of evidence implicating disrupted molecular pathways involved in synaptogenesis and immune function, in addition to a number of molecular mechanisms such as transcription and translation. However, how these observations relate to known inherited mutations in autistic candidate genes, or how disruptions of candidate gene's expression may result in these broad molecular changes, is far from clear.

## Non-coding RNA studies in ASD

Remarkably, most of the single-nucleotide polymorphisms associated with ASD in genome-wide association studies have been in intergenic regions or intronic sequences outside the protein-coding DNA.^102^ Despite these results and the increasing recognition of the importance of ncRNAs in the molecular regulation of human brain development, assessment of ncRNAs in ASD has been limited. Moreover, despite the known tissue-specific nature of ncRNA expression, few of the ncRNA studies in autistic samples have been performed on brain tissue specifically.

To date, four studies have profiled miRNA expression in tissues derived from patients with ‘idiopathic' autism. In the only study of autistic post-mortem brain tissue, Abu-Elneel *et al.*^103^ identified 28 differentially expressed miRNAs in ASD cerebellum via quantitative reverse transcription-PCR analysis. A number of microarray studies have assessed for miRNA expression differences in blood lymphocytes of autistic patients as compared with controls, having discovered nearly 100 miRNAs that are abnormally expressed in ASD.^104, 105, 106^ Another study recently described aberrant expression of lncRNAs in autistic frontal cortex and cerebellum that are antisense transcripts to known autism candidate genes.^107^ This work supports a small study showing lncRNAs are differentially expressed in ASD post-mortem prefrontal cortex and cerebellum, and that previously reported patterning deficits of the mRNA component of the transcriptome in autistic brain are also apparent among lncRNAs.^108^

In addition, recent work has shown that at least 40% of loci previously implicated in ASD abundantly express non-coding antisense transcripts, that these transcripts are often expressed in brain-region-specific manners during development and some are abnormally expressed in autistic post-mortem brain tissue.^107^ Similarly, an earlier study identified a highly significant single-nucleotide polymorphism enriched among ASD patients through genome-wide association analysis, and found it resides in a gene poor locus on chromosome 5p14.1. Further analysis demonstrated that this locus produces an antisense lncRNA to a pseudogene of the mRNA Moesin, and that this lncRNA binds to Moesin in cells, can alter its expression and that Moesin is significantly differentially expressed in post-mortem brain tissue from autistic patients.^109^ Important studies such as these are beginning to link variations found at the DNA sequence level to ncRNA expression differences demonstrated in post-mortem autistic brain tissue.

Therefore, although it appears that ncRNAs are abnormal in ASD patients, their expression and function during human brain development has not been thoroughly characterized, and apart from a few studies attempting to identify mis-expressed ncRNAs, no work has functionally demonstrated how ncRNAs may ultimately contribute to the ASD phenotype. Consequently, there is a critical need to assess for changes in miRNA and lncRNA expression in autistic brain tissue more thoroughly, and to attempt to determine how these changes affect the development of ASD.

## Network and pathway studies in ASD

Finally, several pathway analyses have been performed using either genetic or transcriptome data to gain insight into the biological functions associated with ASD candidate genes. For instance, O'Roak *et al.*^110^ analyzed protein interaction networks among genes implicated in ASD via whole-exome sequencing studies, and identified that *de novo* mutations in ASD patients are over-represented among proteins involved in a chromatin-remodeling network and the β-catenin pathway. Similarly, Gilman *et al.*^111^ demonstrated that copy number variants identified in autistic patients are enriched for genes involved in a molecular network related to synaptogenesis, axon guidance and neuronal motility. These studies represent some of the most rigorous autism cohort sequencing studies performed to date, and their identification of epigenetic processes related to mRNA and ncRNA expression further highlights the importance of this area of research in understanding the molecular mechanisms underlying ASD.^103^

A number of recent studies have attempted to explore the potential functional consequences of autism candidate genes by integrating them with neurodevelopmental gene expression.^112^ Ben-David and Shifman^113^ attempted to assess for differences between rare and common ASD candidate genes by studying their co-expression relationships in adult human brain. They discovered both sets of ASD candidate genes were related to synaptogenesis and neuronal plasticity, and that they are expressed in areas associated with learning, memory and sensory perception.^112^ The same authors also analyzed the neurodevelopmental expression of ASD candidate genes that had been discovered as *de novo* mutations, and demonstrated that these genes relate to molecular networks involved in transcriptional regulation and chromatin remodeling.^113^

Using the same neurodevelopmental transcriptional data set, two other complementary reports recently attempted to infer common biology among various subsets of ASD candidate genes. In the first study, the authors assessed the co-expression relationships among ASD candidate genes they determined to be ‘high confidence' candidates. They demonstrated that these genes are specifically highly co-expressed during mid-fetal development, and in layer 5/6 cortical projection neurons.^114^ The companion study assessed a broader list of ASD candidates, and demonstrated a convergence upon pathways involved in transcriptional regulation during early development, and in synaptogenesis during later childhood.^115^ Although these studies rely on correlative measures to arrive at their conclusions, they provide some of the first rigorous evidence that ASD candidate genes may relate to each other through shared gene expression modules.

## Conclusion and Future Directions

The human brain transcriptome displays a remarkably complex array of mRNAs and ncRNAs that are both temporally and spatially specific. Furthermore, comparative genomics studies have shown that the functional genomics of human brain development diverges significantly from model organisms or even close evolutionary relatives. NcRNAs such as miRNAs and lncRNAs are increasingly recognized as critical regulators of gene expression, and the analysis of gene interaction networks allows for the identification of emergent properties among large sets of genes and ncRNAs that are often not apparent when studying individual genes of interest in isolation. Therefore, it is critical that the genes implicated in autism be understood in the specific context of human neurodevelopmental gene expression, that potential critical ncRNA regulators of their expression be identified and that their network-level properties be explored, as the appropriate temporal–spatial-regulatory context is likely very important to complex neurodevelopmental disorders.

Although studies of ncRNAs in human brain development and autism hold much promise for significantly advancing the understanding of these complex genetic–epigenetic–environmental processes, the field as a whole is still very much in its infancy and there remains a tremendous amount of research to be undertaken before a comprehensive understanding of how ncRNAs help modulate brain development and influence neurodevelopmental disorders can be attained. A number of exciting new areas of research should help to quickly advance this work in the near future, notably through assessment of *in vitro* cell and tissue systems that accurately mimic human brain functional genomics, improved bioinformatics techniques for characterizing ncRNAs and large-scale DNA-sequencing studies of the non-coding regions of the genome in patients with neurodevelopmental disorders.

Foremost, efforts to address the shortage and difficulty of obtaining post-mortem human brain tissue to study ncRNAs are desperately needed if the field is to make significant progress, given the unique human brain-specific patterns of expression previously discussed.^116^ New approaches to modeling human brain development *in vitro* are emerging that may significantly advance this effort, namely from creating neuron-like cells from human tissue as with induced pluripotent stem cell (iPSC) technology. For instance, two recent studies demonstrated that both human embryonic stem cells and murine neural progenitor cells, which were differentiated into neurons, displayed gene expression profiles that were representative of post-mortem human brain tissue.^117^ In addition, iPSCs have been generated from patients with monogenic neurodevelopmental syndromes such as Angelman and Fragile X, potentially allowing for the assessment of disease-specific patterns of ncRNA expression.^118^ Although the use of iPSCs presents advantages for studying native human tissue, there remains concern that the expression and epigenetic signatures of the derived neuron-like cells will more closely mimic the native cell lineage, necessitating continued improvement and expansion of such approaches.^119, 120^ One potential emerging method is the generation of ‘cerebral organoids,' which use stem cell technology to create three-dimesional culture systems that more closely resemble native brain tissue.^121^ Future efforts such as these to advance *in vitro* model systems to more closely mimic human brain tissue expression patterns will be critical to advance the field.

Finally, the emergence of more sophisticated bioinformatics tools to move from simply cataloging to analyzing the vast and complicated ‘ncRNA-ome' is a future step that will be necessary to better interpret signal from noise. One emerging area of research is RNA structure prediction analysis, which can be used to investigate the structure–function relationships of both ncRNAs and disease-causing single-nucleotide polymorphisms via comparative nucleotide sequence analyses, and thereby predict potential functions and/or disease implications of identified ncRNAs.^122^ Similar efforts to move from pure identification of ncRNAs to functional interpretations of their mechanisms of action will be critical, particularly as whole-transcriptome RNA-seq becomes more prevalent.

In summary, the transcriptome of the developing human brain represents a nexus of genetic and environmental interaction that has only recently begun to be rigorously mapped. In particular, it has become clear that non-coding regions of the genome and gene–gene interactions encode layers of information that are of unique importance to human development, to brain development and non-additively to the developing human brain in particular. Although this field is still emerging, already there is evidence that alterations in gene network and ncRNAs may partially underlie common neurodevelopmental disorders. As more studies of gene and ncRNA expression are performed in human post-mortem brain tissue and improving model systems, this area of research is likely to help explain how genes and the environment interact to shape brain development.
